# Long-term psychosocial impact reported by childhood critical illness survivors: a systematic review

**DOI:** 10.1111/nicc.12049

**Published:** 2013-10-23

**Authors:** Joseph C Manning, Pippa Hemingway, Sarah A Redsell

**Affiliations:** JC Manning, RN (Child), PGCert Paediatric Critical Care, MNursSci (Hons), Research Fellow, School of Health Sciences, Faculty of Medicine & Health Sciences, The University of Nottingham, Nottingham, UK; P Hemingway, RGN, RSCN, BSc (Hons), PhD, Lecturer in Nursing Children and Young People, School of Health Sciences, Faculty of Medicine & Health Sciences, The University of Nottingham, Nottingham, UK; SA Redsell, RGN, BSc (Hons), PhD, Professor of Public Health, Faculty of Health, Social Care and Education, Anglia Ruskin University, Cambridge, United Kingdom

**Keywords:** ICU follow-up, Paediatric intensive/critical care, Psychological care, Psychological issues during and after discharge from intensive care, Qualitative research, Short- and long-term patient outcome from intensive care

## Abstract

**Aim:**

To undertake a qualitative systematic review that explores psychological and social impact, reported directly from children and adolescents at least 6 months after their critical illness.

**Background:**

Significant advances in critical care have reduced mortality from childhood critical illness, with the majority of patients being discharged alive. However, it is widely reported that surviving critical illness can be traumatic for both children and their family. Despite a growing body of literature in this field, the psychological and social impact of life threatening critical illness on child and adolescent survivors, more than 6 months post event, remains under-reported.

**Data sources:**

Searches of six online databases were conducted up to February 2012.

**Review methods:**

Predetermined criteria were used to select studies. Methodological quality was assessed using a standardized checklist. An adapted version of the thematic synthesis approach was applied to extract, code and synthesize data.

**Findings:**

Three studies met the inclusion criteria, which were all of moderate methodological quality. Initial coding and synthesis of data resulted in five descriptive themes: confusion and uncertainty, other people's narratives, focus on former self and normality, social isolation and loss of identity, and transition and transformation. Further synthesis culminated in three analytical themes that conceptualize the childhood survivors' psychological and social journey following critical illness.

**Conclusions:**

Critical illness in childhood can expose survivors to a complex trajectory of recovery, with enduring psychosocial adversity manifesting in the long term. Nurses and other health professionals must be aware and support the potential multifaceted psychosocial needs that may arise. Parents and families are identified as fundamental in shaping psychological and social well-being of survivors. Therefore intensive care nurses must take opportunities to raise parents' awareness of the journey of survival and provide appropriate support. Further empirical research is warranted to explore the deficits identified with the existing literature.

## BACKGROUND

More than 250 000 children annually in the USA and the UK require admission to a paediatric intensive care unit (PICU) for the treatment of critical illnesses (Odetola *et al.*, 2005; PICANet, 2011). Over the past four decades there has been a significant reduction in mortality from childhood critical illness due to advances within critical care (Nelson and Gold, 2012). This has resulted in the majority of children and adolescents that are admitted to PICU being discharged alive (≥96%) (PICANet, 2011).

However, critical illness is a significant life event that can cause residual physical and psychological morbidity (Cutler *et al.*, 2013). Childhood critical illness has been reported to be traumatic for survivors and their families with the psychological impact having been extensively explored in the literature (Colville, 2002, Shudy *et al.*, 2006, Rennick and Rashotte, 2009). However, existing research has mainly focused on quantifying psychological impact, immediately after the critical illness and up to 12 months post event. This research is dominated by parents, health professionals and researchers in defining and reporting psychological impact on behalf of childhood survivors. Therefore existing findings may only partially represent the true impact endured by such children and adolescents. Existing research has also predominantly focused on single aspects of impact such as negative emotions, stress or physical impairment. This has resulted in the interplay between the psychological adversity that may be encountered by survivors and the impact on their social world being unreported. This has collectively contributed to a lack of understanding of childhood survivors' self-reported psychological and social impact, experiences, views, perceptions and needs. Clearly, greater understanding of the impact of surviving critical illness is warranted through a review and synthesis of existing qualitative literature.

## RESEARCH STRATEGIES

### Aims

To identify and synthesize qualitative research that explored psychological and/or social impact and needs, as reported by child and adolescent survivors, at least 6 months after their critical illness.

### Design

We conducted a systematic review of primary, qualitative, English language studies informed by Centre for Reviews and Dissemination (2009) guidance. An adapted version of the thematic synthesis method (Thomas and Harden, 2008) was used with a view to enabling the creation of novel themes, explanations or hypotheses.

This review ascribed to a constructivist epistemology where knowledge was seen as being generated in the social world, through the interaction between the researcher and the researched (Bailey, 1997). A realist ontology was adopted that gains access to an underlying reality through the synthesis of varied and multiple perspectives, which is not apparent on the examination of individual accounts or papers (Walsh and Devane, 2012).

### Search methods

**Figure 1:**
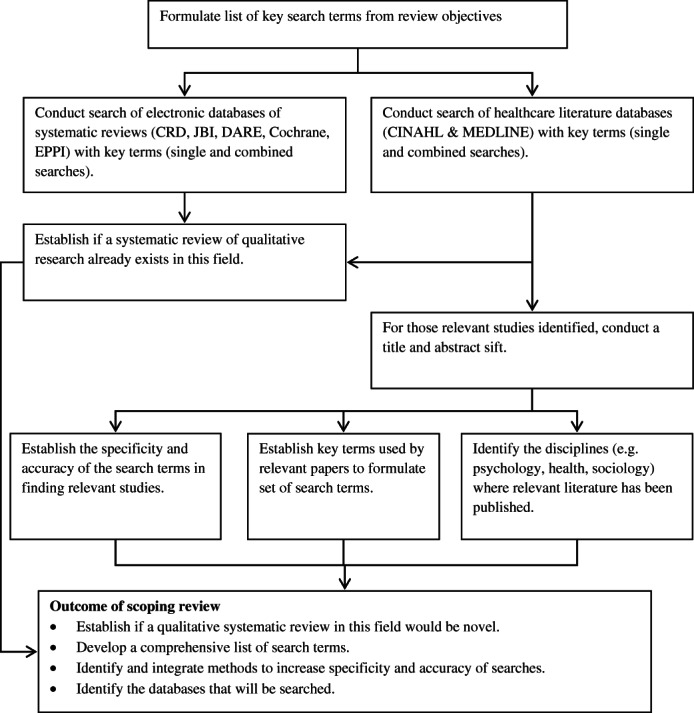
Scoping review flowchart.

An initial scoping review (outlined in Figure 1) conducted in November 2011 did not identify any relevant systematic reviews of qualitative studies. However, scoping did assist in the identification of search terms (Table1) which were refined through discussion with a sociologist and an information scientist.

**Table 1:** Search terms

Adaptation, Psychological
Adolescence
Adolescent Psychology
Adolescent, Hospitalized
Child
Child Psychology
Child, Hospitalized
Critical Illness
Experience
Intensive Care Units, Pediatric
Life threatening
Near Death Experience
Needs
Psychosocial
Social adjustment
Social behaviour
Social identity
Survive
Survivors

In February 2012, electronic databases (CINAHL, MEDLINE, PMC, PsychINFO, ASSIA and Dissertation & Thesis Database) were searched, using single and combination term sets (Table1), to identify relevant studies. Hits were exported into EndNote X5™ following removal of duplicate citations.

The title and abstract of all articles were screened for eligibility using eight inclusion criteria: (1) qualitative primary research, (2) English language, (3) sample studied must include participants that experienced critical illness aged ≤18 years, (4) participants survived an acute life threatening event requiring critical care, (5) outcomes reported by participants children, adolescents and young people, (6) outcomes include psychosocial, psychological, social impact or needs, (7) outcomes reported ≥ 6 months after critical illness and (8) able to extract data from studies with mixed data (such as parent/child reports, critical illness survivors/non-critical illness survivor reports).

Full text copies of studies that satisfied the inclusion criteria were sought. Reference lists were reviewed to identify any relevant studies and key authors were contacted. All studies identified as eligible were then re-screened by two independent reviewers. On the occasions that agreement was not gained, a third reviewer was consulted to achieve consensus.

### Search outcome

**Figure 2:**
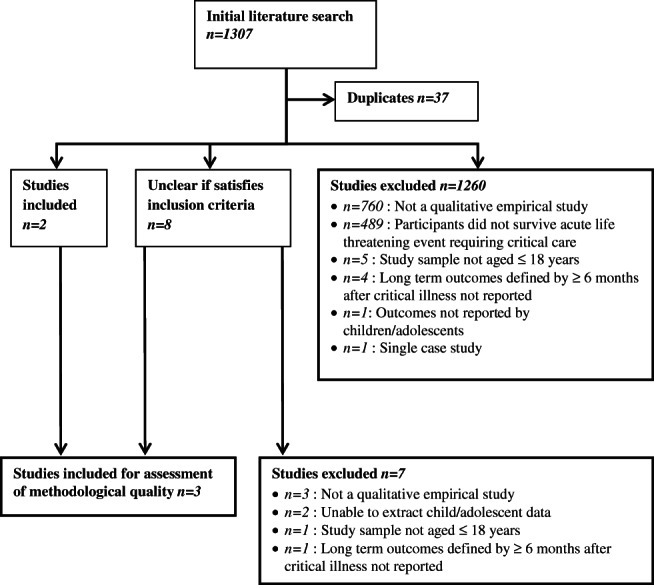
Flowchart of study selection.

The search yielded 1307 citations, with 37 duplicates (Figure 2). Title and abstract screening deemed 1260 studies ineligible, resulting in ten potential studies for inclusion. Full text reports were obtained and further screening resulted in seven of the ten papers being excluded. No additional information was identified from reference lists or contact with study authors. Three studies (Noyes, 2006, Wallace *et al.*, 2007, Atkins *et al.*, 2012) were eligible for assessment of methodological quality.

### Quality appraisal

Two reviewers assessed methodological quality independently using two established quality checklists (by Thomas *et al.*, 2003, Walsh and Downe, 2006). Study characteristics and assessment of methodological quality is summarized in Table2.

**Table 2:** Summary of studies included in review

Study reference/origin	Scope and purpose	Design, methods	Sampling strategy participants	Analytic strategy	Quality rating
Atkins *et al.* (2012), UK	‘To investigate how families came to understand their recovery following their child's admission to PICU’ A range of directly relevant literature and theoretical literature cited. However, the location or strategy not reported.	Grounded theory No discussion of epistemological/ontological grounding Rationale/appropriateness not explored for specific qualitative method Audio tape recorded semi-structured interviews (only with parents in *n* = 4/9 cases)	Purposive sampling used however no justification given. Children and adolescent survivors (*n* = 5) from a single PICU and their carers (*n* = 12). Inclusion criteria: child aged 5–16 at admission; admitted to PICU within the past 8–18 months; child had at least one biological parent participating in the study.	Emergent, iterative, using constant comparative analysis based on Paynes (2007) grounded theory. Unclear how the data was managed and how the context of the data was maintained during the analysis. No evidence of more than one researcher being involved in analysis. However, participant verification of findings noted. Not clear if data saturation was reached. Context described and taken account of in interpretation. However, the use of field notes/interview quotes in discussion of findings were limited. Minimal researcher reflexivity reported. Evident that research was sensitive to ethical concerns.	**C**
Noyes (2006), UK	Experiences and meanings of ventilator-dependent children's health and quality of life Directly related literature cited. However, the location or strategy not reported.	Heideggerian phenomenology Two phase study: phase 1 exploratory qualitative study; phase 2 case studies. Audio taped interviews (only with parents in *n* = 18/53 cases)	Purposive sampling used and justified. Children and adolescent on ventilators (*n* = 35) with 18 as a result of spinal/head injury. Inclusion criteria: aged 1–18; required assisted ventilation for up to 24 h per day; their parents.	Thematic analysis using Heidegger's hermeneutic circle. Data were managed using Atlas Ti™. Not clear if data saturation was reached. No evidence that participants verified findings/interpretations. Minimal researcher reflexivity reported. Evident that research was sensitive to ethical concerns.	**C**
Wallace *et al.* (2007), UK	Experience and impact that altered appearance has for survivors of meningococcal sepsis. Directly related literature cited. However, the location or strategy not reported.	Phenomenology No discussion of epistemological/ontological grounding Audio taped interviews	Purposive sampling used however no justification given. Adolescents and young people. (*n* = 11). Recruited from a national database. Inclusion criteria: aged 10–25; experienced meningococcal sepsis 8 months to 7 years previously.	Interpretative Phenomenological Analysis (IPA) used. Data were analysed and managed by hand. No evidence of more than one researcher being involved in analysis. Not clear if data saturation was reached. No evidence that participants verified findings/interpretations. Demonstrates sensitivity to ethical concerns.	**C**

PICU, paediatric intensive care unit.

### Data abstraction

To ensure that the child and adolescents' accounts were clearly represented, and were not dominated by the interpretations of the reviewers, a data driven method was used. Participant quotes were extracted simultaneously by two independent reviewers which were compared and cross-checked to ensure accuracy and to minimize bias. The extracted data were tabulated for thematic synthesis to be undertaken.

### Synthesis

The thematic synthesis approach utilized an ‘overlapping’ three stage process to compare and translate findings into a higher order (Thomas and Harden, 2008). Free line-by-line coding was conducted by two reviewers independently and then organized into related groups called ‘descriptive themes’. These were independently verified by a third reviewer who was given opportunity to add additional codes and ‘descriptive themes’. These ‘descriptive themes’ were then analysed and synthesized, which involved cross translation, to form ‘analytical themes’.

## FINDINGS

### Study description

All studies were conducted in the UK involving a total of 51 survivors. Studies used purposive sampling, with participants having experienced critical illness due to a range of conditions (predominantly head/spinal injury, sepsis or malignancies). Semi-structured interviews were the main form of data collection.

### Data coding

Twenty participant quotations were extracted (Atkins *et al.* 2012, (*n* = 7); Noyes 2006, (*n* = 6); Wallace *et al.* 2007, (*n* = 7)). Initial coding identified 81 codes which were verified by the third reviewer who proposed five additional codes, resulting in 86 codes in total.

### Descriptive themes

**Figure 3:**
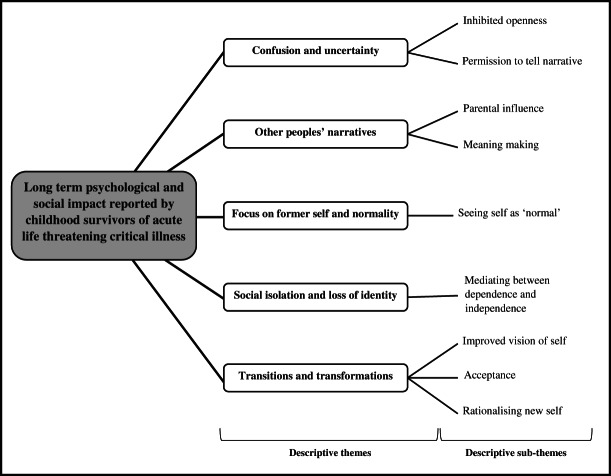
Thematic synthesis of descriptive themes and sub themes.

From these 86 codes, nine sub-themes and five major descriptive themes (Figure 3) were developed.

#### Theme 1: confusion and uncertainty

Up to 1 year after their critical illness participants, particularly adolescents, indicated complexity and confusion surrounding their life events. When requested to report their critical illness experience, participants replied with uncertainty. Responses ranged from forgotten or unknown experiences, such as ‘I don't remember much …’ (Atkins *et al.*, 2012. p.137) or ‘it was like a chapter of my life had been taken out’ (Atkins *et al.*, 2012. p.137), to demonstrating difficulty in constructing and articulating what had happened to them, such as ‘I would like to but it's hard to explain it and stuff …’(Atkins *et al.* 2012. p.137). Participant responses could have been influenced by a multitude of factors such as familiarity with the researcher and embarrassment, researcher-participant parity, and whether there were other people present (e.g. parents) during the interview. However, confusion and uncertainty were also evident in reports of participants' sharing their critical illness story with other people, such as friends and family members. Participants displayed a reluctance to share their experiences with other people from their social group. This inhibited openness appeared to emanate from feelings of anxiety or embarrassment relating to a lack of understanding surrounding their critical illness. Inhibitions also transpired from participants who demonstrated some understanding of their critical illness experience. However, these participants' uncertainties emanated from their accounts conflicting with the experiences and narratives of others (e.g. parents). Collectively, this confusion and uncertainty provoked a level of awkwardness in participants and subsequently manifested in them requiring permission or approval from other people prior to disclosing their own experiences, views and narratives.

#### Theme 2: other peoples' narratives

From confusion and uncertainty, participants described how they attempted to understand or gauge a level of clarity through the narratives of others, particularly parents and relatives. Other peoples' narratives were also used by participants to make sense of their situation allowing them to locate their new self and social identity following critical illness. Parental narratives appeared to be most significant in forming and influencing participants' perspectives and views of their critical illness experience and the life events that surrounded it.

#### Theme 3: focus on former self and ‘normality’

Participants placed an overwhelming emphasis on their former self, a position which they viewed as ‘normal’. They perceived that some families and friends found the person they were prior to the critical illness experience more acceptable than the person they had become. This led them to desire a return to their former self. For many participants this was unrealistic as they had experienced a physical change such as altered appearance or dependence on mechanical ventilation.

However, the focus on ‘normality’ provided participants with a drive to assume roles and routines they had undertaken prior to being critically ill. In many cases parents were instrumental in directing the participants' aspirations to retain their former self. However, this emphasis may have been detrimental to participants overall well-being as participants reported being unable to achieve what would previously have been normal for them. Thus resulting in a realization of failure and negative emotions.

#### Theme 4: social isolation and loss of identity

Participants described feelings of isolation and abandonment following their critical illness. These feelings emerged from fluctuating social identities that reflected changes to the participants' identity, dependence and ability. Following their critical illness participants indicated that they were perceived as a novelty in relation to their peers. Interest in participants was initially present but later subsided, thus leaving survivors feeling lonely and rejected.

Participants reported a dependency particularly on parents for physical assistance and the maintenance of health. However, a discourse of conflict between independence and this newly realized dependency was purported. This was particularly apparent in adolescent accounts and could be attributed to dependence on others inhibiting their ‘normal’ personal and social developmental growth trajectory. In response, participants described conflicts when negotiating independence which resulted in emotions such as frustration.

#### Theme 5: transition and transformation

The final descriptive theme encompassed the transitions of self and social identity. Participants experienced transformations that did not assume a linear trajectory as recurrent periods of change and adjustment were reported.

For some transformations emanated from a confused or forgotten state where participants' self-identity was unclear due to changes in physical health and social status. This rendered some survivors into a state of disequilibrium. Participants searched for meaning and understanding of their current state by collecting information from others. A period of adjustment then followed enveloped by the aforementioned themes but also warranted time for the participant to reflect and rebuild their self and social identity.

As part of the rebuilding and adjustment process, participants reported a level of acceptance in relation to their new identity. However, in conjunction with this acceptance, the focus of participants' narratives and experiences altered. Participant dialogue changed from constant comparison to their ‘normal’ former self to an acceptance of their new identity. During this transformation period participants reflected on their current status, rationalizing their experience and subsequent outcomes either in line with what had happened to other people who had experienced similar events or by focusing on what could have happened to themselves. Ultimately participants began to rebuild and mediate a new or adapted self and social identity which encapsulated their journey and current physical, psychological and social status.

### Analytical themes

**Figure 4:**
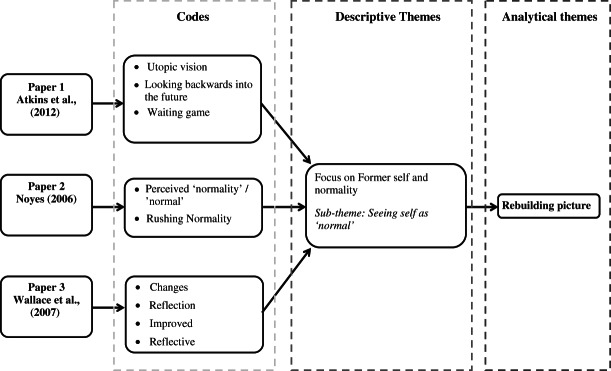
A worked example of the process used to synthesize the analytical theme ‘rebuilding picture’.

The five major themes identified from the descriptive synthesis were subjected to further examination. The original review questions were used to interrogate these themes with an aim of producing higher order themes and defining the relationships between them (Cruzes and Dyba, 2011). The process undertaken to develop the analytical themes is presented in Figure4 using a worked example. Higher order themes included: ‘missing picture’, ‘rebuilding picture’, and ‘new-self and social identity’.

#### Analytical theme 1: missing picture

It is apparent from survivor self-reports that the initial adversity encountered emanated from confusion and ambiguity in relation to their biographical narrative. Lack of clarity surrounding their critical illness experience rendered children and adolescents perplexed and unable to comprehend or share their story. This denied survivors the meanings and explanations (in relation: to their life events; to what had happened to them; and to their current existence) that were required to locate their self and social identities within the world. This period following critical illness was identified as the theme ‘missing picture’ and negatively impacted on the psychological and social status of survivors.

#### Analytical theme 2: rebuilding picture

From this period of ambiguity (‘missing picture’), it was evident that a pursuit to regain control and understanding over the survivors new self subsequently ensued (‘rebuilding picture’). This dynamic phase encompassed a number of processes and influences. Through the collection of other people's narratives, survivors began to piece together and rebuild the missing picture. However, this process was not without on-going adversity. Survivors had to navigate conflicts between their own, and other people's, utopic desires for them to resume their former ‘normal’ self. For many survivors this involved mediating between their expected or perceived abilities and the realities of their new, and potentially altered, being. Rebuilding their self and social identities required realization, adjustment and realignment of survivors' expectations and norms. For some survivors this exposed them to additional adversity. Abandonment, psychological and social isolation was experienced either as a result of the dynamics of the social group not aligning to the survivor or vice versa. Collectively, exposure to this adversity contributed to the realization that the self and social identities were transforming and being redefined.

#### Analytical theme 3: new self and social identity formed or forming

This phase or period of transformation continued until survivors began to accept their newly defined self. For some survivors this was not a finite stage but continued as a dynamic process. This phase also recognized a change from mediating between unachievable expectations, to rationalizing and implementing coping mechanisms to deal with the reality of themselves as a transformed or transforming physical, emotional and social being.

**Figure 5:**
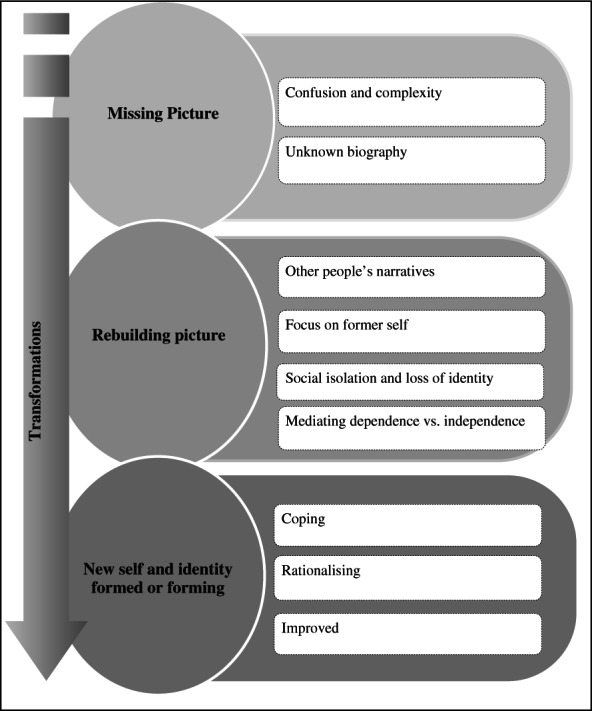
Thematic synthesis conceptual framework.

Collectively, the interplay between these analytical themes is schematically represented in Figure 5.

## DISCUSSION

### Psychological and social adversity

Findings from this review have identified that surviving critical illness can expose survivors to long-term psychological and social adversity and impact. Thematic synthesis has identified that this transpires from biographical disturbances and conflicting identities. However, these findings contrast with existing literature that purports psychological impact prevails from the iatrogenic nature of PICU (Rennick and Rashotte, 2009). Although it cannot be refuted that the aforementioned adversity could be influenced by exposure to PICU, including treatments and medications, it is evident from this review that during long-term survival adversities unfold and manifest that are separate to the direct intensive care experience. This review identified that psychological and social impact can emanate from a ‘missing picture’. Although the utilization of this term is novel, similar themes have been reported in the adult ICU and wider health care literature. Failure to recall a critical illness experience is common in adult ICU patients (Stein-Parbury and McKinley, 2000), emanating from delirium and hallucinations which have been attributed to polypharmacy and physiological changes (Truman and Ely, 2003, Skrobik *et al.*, 2004). A recent review and synthesis of qualitative literature from adult ICU survivor self-reports categorizes this theme under ‘transformations of perception: unreal experiences and dreams’ (Cutler *et al.*, 2013, p.149). Similar to our findings, Cutler *et al.* (2013) purport that survivors experienced an altered sense of reality and perception that transpired into negative emotions and distress. However, this was reported to occur during or immediately after critical illness, with conflicting findings presented as to whether survivors engaged with their critical illness event in the longer term (Cutler *et al.*, 2013). This indicates that despite this phenomenon being evident in both adult and childhood critical illness survivor self-reports, the way in which it manifests appears to differ.

From initial uncertainty, a dynamic process of rebuilding and transformation occurs. The initial stages of this process involve the collection and acquisition of narratives surrounding the critical illness experience. The use of other people's narratives could be explained by Taylor's (1983) Theory of Cognitive Adaption who defines the period post illness as ‘a search for meaning’. Although this theory was constructed from research with female breast cancer survivors, Taylor (1983) identifies that meaning making following a life threatening event is essential to understand the cause and significance of the event. Our findings showed that survivors used the narratives of others to provide a platform to reflect upon their core values and prioritize things that were important to them. Similarly, Cutler *et al.* (2013) identifies that adult survivors also use other people for clarification and meaning making following critical illness. However, in contrast to our findings, health professionals were a significant group of informants for adult survivors and were used for narrative verification, safety and security (Cutler *et al.*, 2013).

Despite the utilization of other people's narratives to provide some elements of meaning and rebuild their picture, survivors continued to endure psychological and social impact. Survivors focussed on their former ‘normal’ self and resisted their new self and social identity. Bury (1982) describes a similar phenomenon as an upset in relation to ‘internal and external realities’ where the survivor encounters a ‘biographical shift’ from that of a perceived normal trajectory to one that is abnormal and possibly ‘inwardly damaging’. This could provide a possible explanation as why PICU survivors place an emphasis on their former being and are resistant to something that is as described as ‘damaging and abnormal’ (Bury, 1982). However, what is not explored by Bury is the impact of resistance as our findings suggest that on-going resistance to the new self and social identity is also damaging. It could be argued that without adjustment the survivor will endure further negative psychological and social adversity.

Charmaz (1983) also provides an interesting insight into the rebuilding of self following illness, specifically chronic illness. She proposes that experiencing illness is a social and psychological process where the inner dialogue between ‘I’ and ‘me’ changes as well as survivors own definitions of experiences. Charmaz (1983) identifies that survivors have a restricted life imposed upon them where values of independence and individualism are challenged. Moreover, survivors are unable to exercise choice in valued activities resulting in self-image not being maintained. However, similar to the rebuilding process identified from the analytical themes, Charmaz (1983) reports that this adversity is the foundation from which re-evaluation and change occurs.

### Deficits in the existing literature

From the papers included in this review it is evident that a number of deficits exist. There is a bias towards the reports of older children/adolescents and children that were able to articulate their experiences verbally. Subsequently this has limited our understanding of the psychological and social impact from the perspectives of young children and those unable to communicate verbally.

Clearly, childhood survivors of critical illness have a number of outstanding needs that manifest in the long term. These include: a need for information, to alleviate any uncertainty or ambiguity surrounding their life in order to reassemble the missing or partially formed picture; a need for time, to grieve for their former self, to explore and understand their experiences (meaning making), and to reflect and rebuild their new self and social identities; and a need to accept, to allow for adaption/adjustment to their new physical, psychological and social reality. However, we (as the reviewers) have identified these needs and not the children and adolescent survivors themselves thus acknowledging that there is a distinct scarcity of child and adolescent reported needs within the existing literature.

### Strengths and potential limitations of the review

Although many hundreds of papers were identified that explored the impact of illness in childhood, very few fulfilled the inclusion criteria for this review. It can therefore be concluded that the search strategy had high sensitivity; however, despite measures implemented to maximize specificity (e.g. search filters) this remained low. This occurrence of a trade-off between sensitivity and specificity is reported within the literature, with highly sensitive strategies having low levels of precision and vice versa (Petticrew and Roberts, 2010). However, for reviews where the ‘intervention’, or as in this study the ‘phenomena of interest’, is difficult to define it is advocated to maximize sensitivity at the risk of specificity to ensure an exhaustive search (Petticrew and Roberts, 2010).

Despite these strengths there are a number of limitations that we must recognize. Owing to the extensive search strategy, any relevant English language research studies should have been identified. However, this is limited to those research studies that were indexed on databases from the Western world. This could have contributed to all studies included being conducted in the UK, thus providing an ethnocentric perspective of psychological and social impact and needs of surviving critical illness in childhood. We recognize that this may limit the transferability of the review findings to other contexts outside the Western world. However, conversely it could be argued that contextual homogeneity is beneficial as included participants have experienced the same health care system, thus strengthening the synthesis and transferability of the findings.

The descriptive and analytical themes in this review are based on participants, from three studies, who experienced ‘life threatening’ critical illness. We acknowledge that children and adolescents who experienced ‘life threatening’ critical illness may be more severely ill than others who survive critical care. Therefore a potential limitation of the review is that the conceptual framework proposed may only reflect survivors who were severely critically ill and not the general PICU populous.

Furthermore, the heterogeneity of the sample in relation to outcomes (physical and health care needs) could be perceived as a limitation of this review. Certain participants had experienced specific conditions and required on-going medical support which could have influenced their psychological and social well-being and needs. However, by synthesizing the reports from multiple survivors, which vary in outcomes and conditions, we hoped to gain access to an underlying reality.

## CONCLUSION

Survival of childhood critical illness can expose patients to a complex array of adversity which emanates from biographical disturbances and the misalignment between an idealistic focus on their ‘normal’ prior self and the realities of their changed being. In the longer term following critical illness, a dynamic process of transformation occurs where survivors attempt to overcome the adversity they face. For some survivors this involves attempting to fill the missing picture, through the acquisition and exploration of other people's narratives. In addition, survivors confront new conflicts that present such as dependence versus independence and the new social identity that has been imposed upon them. It is therefore evident that psychosocial impact as a result of critical illness is not transient with residual adversity and on-going needs manifesting in survivors for some time after the event has occurred.

### Application to nursing practice

Intensive care nurses need to prepare parents and families for the potential adversities that may be faced by survivors following critical illness. Communicating with parents the influence they have in shaping and supporting their child's psychological and social well-being is essential.

Models of nursing care for survivors need to be evaluated to ensure that they do not cause unnecessary psychological harm by focusing on a former self that reinforces unachievable goals. Moreover, nurses and other professionals, from sectors such as health care, social care and education, who come into contact with child and adolescent survivors, need to comprehend and support the potential complex psychological and social trajectory of survival.

### Application to future research

This review has provided an insight into the transformations that children and adolescents may encounter following critical illness. However, the exact trajectory, as well as intrinsic and extrinsic influences as reported by children and adolescents, is unclear. Therefore further empirical research is warranted, especially with younger children and using a range of inclusive methods, that aims to explore and understand this complex and multifaceted phenomenon. In addition, the framework proposed from the analytical themes now warrants exposure to interrogation and refinement through application to empirical enquiry.

## References

[b1] Atkins E, Colville G, John M (2012). A ‘biopsychosocial’ model for recovery: a grounded theory study of families' journeys after a Paediatric Intensive Care Admission. Intensive and Critical Care Nursing.

[b2] Bailey PH (1997). Finding your way around qualitative methods in nursing research. Journal of Advanced Nursing.

[b3] Bury M (1982). Chronic illness as biographical disruption. Sociology of Health & Illness.

[b4] Centre for Reviews and Dissemination (2009). Systematic Reviews: CRD's Guidance for Undertaking Reviews in Health Care.

[b5] Charmaz K (1983). Loss of self: a fundamental form of suffering in the chronically ill. Sociology of Health & Illness.

[b6] Colville G, Ridley S (2002). The impact of admission to paediatric intensive care unit (PICU) on the child and family. Critical Care Focus 12: The Psychological Challenges of Intensive Care.

[b7] Cruzes DS, Dyba T (2011).

[b8] Cutler LR, Hayter M, Ryan T (2013). A critical review and synthesis of qualitative research on patient experiences of critical illness. Intensive and Critical Care Nursing.

[b9] Nelson LP, Gold JI (2012). Posttraumatic stress disorder in children and their parents following admission to the pediatric intensive care unit: a review. Pediatric Critical Care Medicine.

[b10] Noyes J (2006). Health and quality of life of ventilator-dependent children. Journal of Advanced Nursing.

[b11] Odetola FO, Clark SJ, Freed GL, Bratton SL, Davis MM (2005). A National Survey of pediatric critical care resources in the United States. Pediatrics.

[b12] PICANet (2011). Annual Report of the Paediatric Intensive Care Audit Network January 2008–December 2010.

[b13] Petticrew M, Roberts H (2010). Systematic Reviews in the Social Sciences: A practical guide.

[b14] Rennick JE, Rashotte J (2009). Psychological outcomes in children following pediatric intensive care unit hospitalization: a systematic review of the research. Journal of Child Health Care.

[b15] Shudy M, de Almeida ML, Ly S, Landon C, Groft S, Jenkins TL, Nicholson CE (2006). Impact of pediatric critical illness and injury on families: a systematic literature review. Pediatrics.

[b16] Skrobik Y, Bergeron N, Dumont M, Gottfried S (2004). Olanzapine vs haloperidol: treating delirium in a critical care setting. Intensive Care Medicine.

[b17] Stein-Parbury J, McKinley S (2000). Patients' experiences of being in an intensive care unit: a select literature review. American Journal of Critical Care.

[b18] Taylor SE (1983). Adjustment to threatening events: a theory of cognitive adaptation. American Psychologist.

[b19] Thomas J, Harden A (2008). Methods for the thematic synthesis of qualitative research in systematic reviews. BMC Medical Research Methodology.

[b20] Thomas J, Sutcliffe K, Harden A, Oakley A, Oliver S, Rees R, Brunton G, Kavanagh J (2003). Children and Healthy Eating: A Systematic Review of Barriers and Facilitators.

[b21] Truman B, Ely EW (2003). Monitoring delirium in critically ill patients: using the confusion assessment method for the intensive care unit. Critical Care Nurse.

[b22] Wallace M, Harcourt D, Rumsey N (2007). Adjustment to appearance changes resulting from meningococcal septicaemia during adolescence: a qualitative study. Developmental Neurorehabilitation.

[b23] Walsh D, Downe S (2006). Appraising the quality of qualitative research. Midwifery.

[b24] Walsh D, Devane D (2012). A Metasynthesis of midwife-led care. Qualitative Health Research.

